# High quality RNA extraction from Maqui berry for its application in next-generation sequencing

**DOI:** 10.1186/s40064-016-2906-x

**Published:** 2016-08-03

**Authors:** Carolina Sánchez, Javier Villacreses, Noelle Blanc, Loreto Espinoza, Camila Martinez, Gabriela Pastor, Patricio Manque, Soledad F. Undurraga, Victor Polanco

**Affiliations:** Laboratorio de Biotecnología Vegetal, Centro de Genómica y Bioinformática, Universidad Mayor, 8580000 Santiago, Chile

**Keywords:** Maqui berry, CTAB, RNA extraction, RNA-Seq

## Abstract

Maqui berry (*Aristotelia chilensis*) is a native Chilean species that produces berries that are exceptionally rich in anthocyanins and natural antioxidants. These natural compounds provide an array of health benefits for humans, making them very desirable in a fruit. At the same time, these substances also interfere with nucleic acid preparations, making RNA extraction from Maqui berry a major challenge. Our group established a method for RNA extraction of Maqui berry with a high quality RNA (good purity, good integrity and higher yield). This procedure is based on the adapted CTAB method using high concentrations of PVP (4 %) and β-mercaptoethanol (4 %) and spermidine in the extraction buffer. These reagents help to remove contaminants such as polysaccharides, proteins, phenols and also prevent the oxidation of phenolic compounds. The high quality of RNA isolated through this method allowed its uses with success in molecular applications for this endemic Chilean fruit, such as differential expression analysis of RNA-Seq data using next generation sequencing (NGS). Furthermore, we consider that our method could potentially be used for other plant species with extremely high levels of antioxidants and anthocyanins.

## Background

RNA isolation has always been a critical first step in plant molecular biology. The structure of RNA contains a ribose sugars with a 2′-hydroxyl group attached that makes it more sensitive to degradation. This inherent instability together with the high levels of secondary metabolites found in plants hinders the RNA extraction and most of the times the RNA obtained has low concentration and poor quality (Sah et al. 2014).

Next-generation sequencing (NGS) is a high-throughput sequencing technology, extremely sensitive to contamination and requires high quality of nucleic acids (Nadiya et al. 2015). Gene expression studies need to use a sufficient quantity of pure RNA to get accurate results (Yockteng et al. 2013). Therefore, protocols aimed at the purification of copious amounts of high quality RNA are vital for assays using these new methodologies (Yockteng et al. 2013; Nadiya et al. 2015).

There are many commercial kits and protocols for plant RNA extraction using different strategies. However, cannot always be successfully applied to non-model species (Sah et al. 2014). And most of the time is required to test the sample of interest to evaluate if the method works and the results often depend on the organism and tissue studied (Sah et al. 2014). Furthermore, nucleic acid isolation from fruit tissues is particularly challenging due to the high levels of phenolic compounds, polysaccharides and RNases (Jones et al. 1997). Currently, there is no standard protocol for plant RNA extraction and many approaches are used according to the species and tissue used (Nadiya et al. 2015).

In recent years, South American berries have gained increased attention from all over the world. This interest has mainly been driven by their potential health benefits as well as a growing consumer demand for novel exotic fruits (Speisky et al. 2012). Among these berries, the Chilean Maqui fruit (Maqui berry; *Aristotelia chilensis* [Mol.] Stuntz, Elaeocarpaceae) has been singled out as an exceptionally rich source of anthocyanins and natural antioxidants (Speisky et al. 2012). Maqui berries were reported to have much higher total polyphenolic content and antioxidant activity than other polyphenol-rich fruit species, such as blackberries (*Rubus* spp.), blueberries (*Vaccinium* spp.), raspberries (*Rubus idaeus* L.) and strawberries (*Fragaria* × *ananassa* L.) (Kähkönen et al. 2001; Miranda-Rottmann et al. 2002). Despite their antioxidant benefits, these natural compounds interfere with nucleic acid preparations, making RNA extraction a major challenge (Lorenz et al. 2010; Ouyang et al. 2014). The phenolic substances trigger oxidation and degradation because irreversibly bind to proteins and nucleic acids.

In order to extract RNA from Maqui berry, our group tested unsuccessfully several published protocols including methods developed from other fruits with high levels of polyphenols and berries closely related to Maqui berry (Hughes and Galau 1988; John 1992; Boss et al. 1996; Jones et al. 1997; Woodhead et al. 1997). Therefore, the aim of this study was to develop one simple and efficient RNA extraction protocol of Maqui berry with high quality: good purity, good integrity and higher yield by adapting the procedure developed by Jaakola et al. 2001. Our method is based on the use of an extraction buffer containing double concentration of polyvinylpyrrolidone (PVP) and β-mercaptoethanol. PVP removes phenolic compounds and secondary metabolites from nucleic acid preparations, and it also prevents browning effect of polyphenols. β-mercaptoethanol is an antioxidant/reducing agent that irreversibly denature RNases by reducing disulfide bonds and destroying the native conformation required for enzyme functionality. The RNA obtained had sufficient quality for RNA-Seq library construction. We successfully accomplished this goal by adapting a total RNA extraction protocol for bilberry (Jaakola et al. 2001).

## Results and discussion

Isolation of high quality RNA from non-model plants, such as Maqui berry is a challenge due to high levels of polyphenols, anthocyanins, polysaccharides, and other types of secondary metabolites present in the sample.

We tried to isolate RNA using several commercially available RNA extraction kits based in silica columns unsuccessfully. The samples obtained had no absorbance at A260 nm and they could not be visualized; it might be caused by the release of high contents of polyphenolics and metabolites after disruption of the cells, which are embedded in viscous polysaccharides during RNA extraction.

On the other hand, we assayed various plant RNA extraction protocols for species and tissues with high levels of phenolic compounds and polysaccharides based on hexadecyltrimethyl ammonium bromide (CTAB), including the one developed by Jaakola et al. 2001. However, these methods did not allow us to obtain RNA for subsequent experiments.

**Fig. 1:**
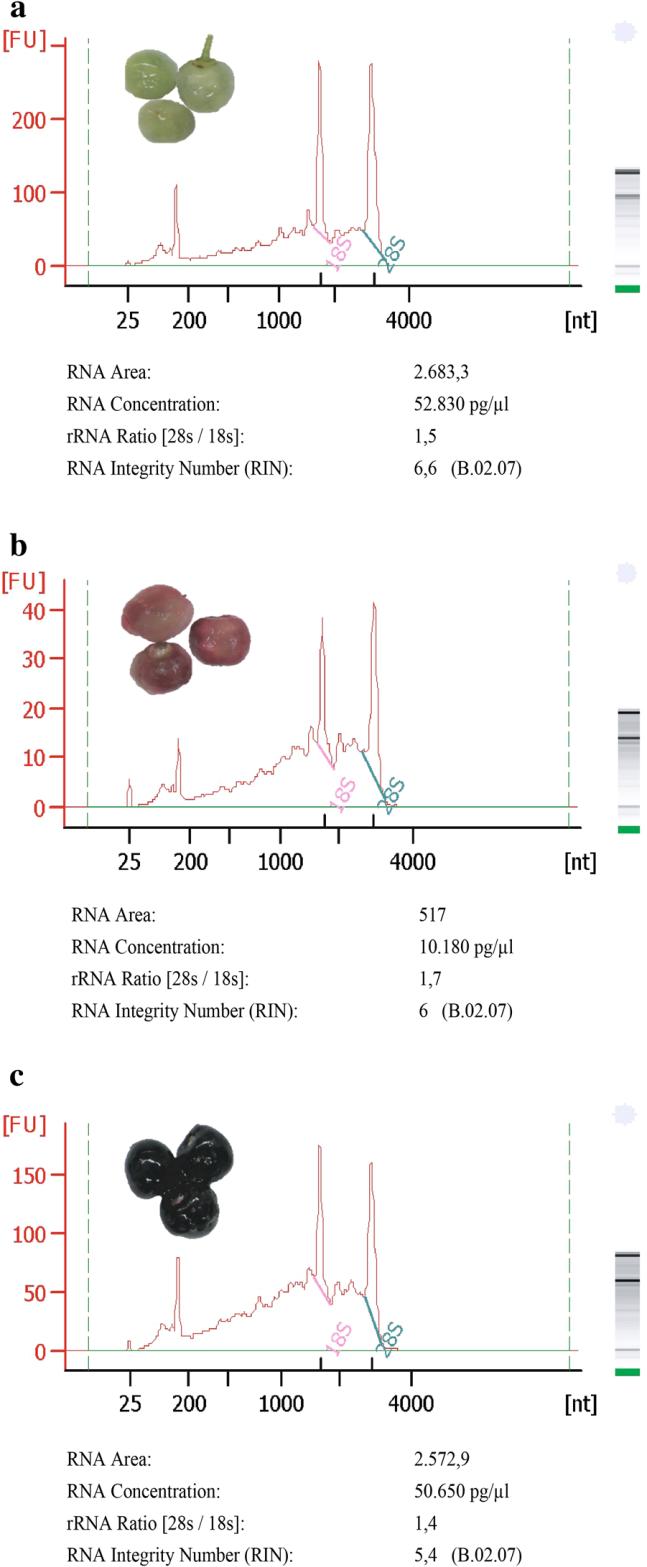
Electrophoretogram of total RNA obtained with our new method. The 18S and 28S rRNA 25 regions are shown. RNA concentrations and RIN values are shown below. **a** green fruit. **b** red fruit. **c** blue fruit. RNA was analyzed with the Agilent RNA 6000 Nano Assay in a 2100 bioanalyzer (Agilent Technologies). Note that the output from our instrument is completely opposite from the English convention, therefore, it uses commas as an indicator of decimals, and periods to denote thousands. It is shown one result of the three RNA extraction obtained from the same tissue

To overcome these problems and to get cleaner samples, many authors have proposed the addition of contaminant absorbents like PVP in combination with a powerful reductant (β-mercaptoethanol) into the extraction buffer (Jaakola et al. 2001; Liao et al. 2004; Wang et al. 2005). We developed a method which it is an adaptation of Jaakola protocol resulting in RNA preparations with high quality (Fig. 1).

This protocol was based on a CTAB extraction buffer supplemented with high amounts of PVP and β-mercaptoethanol to properly remove polysaccharides and prevent oxidation of phenolic compounds. In our experiments, high concentrations of β-mercaptoethanol (4 %) and PVP (4 %) present in the extraction buffer prevented the oxidation of phenolic compounds, which were subsequently removed using organic solvents. The concentration of the absorbent component (PVP) was higher than those reported for other berries (2–3 %, W/V) (Jaakola et al. 2001; Liao et al. 2004; Wang et al. 2005). Furthermore, spermidine, a polyamine that binds nucleic acids, was added to the extraction buffer in order to compete for RNA binding sites, competing against any of the remaining polyphenol molecules (Leipold 1977).

The LiCl precipitation and the use of acid phenol (pH ~4.3) in phenol–chloroform extraction were done to specifically remove DNA. These steps allowed the recovery of RNA with low levels of DNA contamination. The 70 % ethanol wash was essential to ensure RNA purity and, with ripe fruits, this step had to be repeated twice or three times, successfully eliminating the colorful anthocyanin pigments, which can interfere with subsequent steps.

A summary of the steps of RNA extraction method it is shown in Table 1 and the function of the main components in Table 2.

**Table 1:** Steps of RNA extraction method from Maqui berry

Steps of RNA extraction method	
1. Cell lysis	Grind 1.5 g fresh tissue to fine powder with a mortar and pestle under liquid nitrogen
	Add 750 μl of preheated extraction buffer (65 °C)
	Incubate at 65 °C for 10 min and agitate with a vortex for a few seconds during the incubation time
	Centrifuge at 10,000×*g* (RCF) for 10 min at 4 °C
	Recover the supernatant and mix with an equal volume of chloroform:IAA
	Mix and centrifuge at 15,700×*g* (RCF) for 10 min at 4 °C
2. RNA precipitation	Recover the supernatant and add 1/4 volume of 10 M LiCl. Mix gently
	Precipitate the RNA at −80 °C overnight
	Centrifuge at 18,000×*g* (RCF) for 20 min at 4 °C
	Wash the pellet with 500 µL of 70 % ice-cold ethanol
	For ripe fruits, repeat this washing step at least twice, until the dark color disappears
	Centrifuge briefly and remove the 70 % ethanol
	Dissolve the RNA pellet in 100 µl of SSTE buffer. Briefly heat at 65 °C if required
3. RNA clean up	Add equal volume of phenol:chloroform:IAA (25:24:1) to the sample and shake vigorously
	Incubate on ice for 5 min
	Centrifuge at 18,000×*g* (RCF) for 20 min at 4 °C
	Carefully transfer the supernatant to a new tube
	Add equal volume of chloroform:IAA
	Centrifuge at 18,000×*g* (RCF) for 20 min at 4 °C
	Transfer the upper aqueous phase to a new tube
4. RNA precipitation	Precipitate RNA by adding an equal volume of absolute ethanol
	Incubate at −20 °C for 2 h or at −80 °C for 30 min
	Centrifuge at 18,000×*g* (RCF) for 15 min at 4 °C
	Discard the supernatant
	Carefully wash the pellet twice with chilled 70 % ethanol
	Dry the pellet and dissolve in 200 μl DEPC-treated water

Extraction buffer: 2 % CTAB, 4 % polyvinylpyrrolidone (PVP-40), 100 mM Tris–HCl (pH 8.0), 25 mM EDTA and 2.0 M NaCl. Add spermidine and β-mercaptoethanol to a final concentration of 0.5 g/l and 4 %, respectively, just before use. SSTE buffer: 1.0 M NaCl, 0.5 % SDS, 10 mM Tris–HCl (pH 8.0), 1 mM EDTA (pH 8.0)

**Table 2:** Function of the compounds used in the RNA extraction method from Maqui berry

Compound	Function
Hexadecyltrimethyl ammonium bromide (CTAB)	This detergent simultaneously solubilizes the plant cell wall and lipid membranes of internal organelles and denatures proteins (enzymes)
PVP-40	Remove phenolic compounds and secondary metabolites from nucleic acid preparations, and it also prevent browning effect of polyphenols
Spermidine	Binds to and precipitates nucleic acid
β-Mercapoetanol	An antioxidant/reducing agent that will irreversibly denature RNases by reducing disulfide bonds and destroying the native conformation required for enzyme functionality
Lithium chloride	Allows the precipitation of RNA because it does not efficiently precipitate either protein or DNA

Table 3 shows the A260/A280 ratio for the samples. The 260/280 values of total RNA from various tissues ranged from 1.9 to 2.0, indicating a high purity of the obtained RNAs. Although A260/A280 ratio is an important indicator of sample quality, the best indicator of RNA quality is functionality in the downstream applications.

**Table 3:** Average yields, A260/A280 and A260/A230 ratios of isolated RNA from different tissues of Maqui berry

Tissue	Yield (ng/μL)^a^	Bioanalyzer concentration (ng/ul)^a^	260/280 ratio^a^	260/230 ratio^a^
Green fruit	132.75	52.8	2.19	1.86
Red fruit	26.47	10.1	1.99	1.66
Blue fruit	116.49	50.7	2.13	1.56

^a^Average of three extracts from the same tissue

RNA integrity from our Maqui berry preps was evaluated with the 2100 Bioanalyzer instrument using the Agilent RNA 6000 Pico Assay Kit (Agilent Technologies). The RNA profiles obtained by this method allow an accurate assessment of RNA integrity by ribosomal integrity number (RIN) values (Fig. 1). All of our samples gave RIN values higher that five, representing good RNA quality for downstream applications (Schroeder et al. 2006). Our figures show peaks produced from electrophoretograms that depict the size distribution of RNA fragments, the corresponding gel-like image of RNA fragments, and metrics of RNA concentration and integrity (r26S/18S and RIN). Concentrations for the 26S and 18S ribosomal RNAs were calculated by taking the area below the green and pink straight lines at the base of the 26S and 18S peaks, respectively. Figure 1 shows that total RNA of green fruit (A), red fruit (B) and blue fruit (C), exhibited only trace levels of polyphenols and polysaccharides. This indicates that the RNA is relatively free of contamination. As a result, we recovered total RNA that ranged between 6 and 18 µg per gram of fresh weight of starting material. This gave final RNA concentrations between 10 and 53 ng/µl (10.180 and 52.830 pg/µl, respectively (Fig. 1). The bioanalyzer quantification is more reliable than those obtained by spectrophotometric method (Table 3). The contaminants that absorb around 260 nm can contribute to the absorbance value, resulting in an overestimation of RNA concentration.

**Fig. 2:**
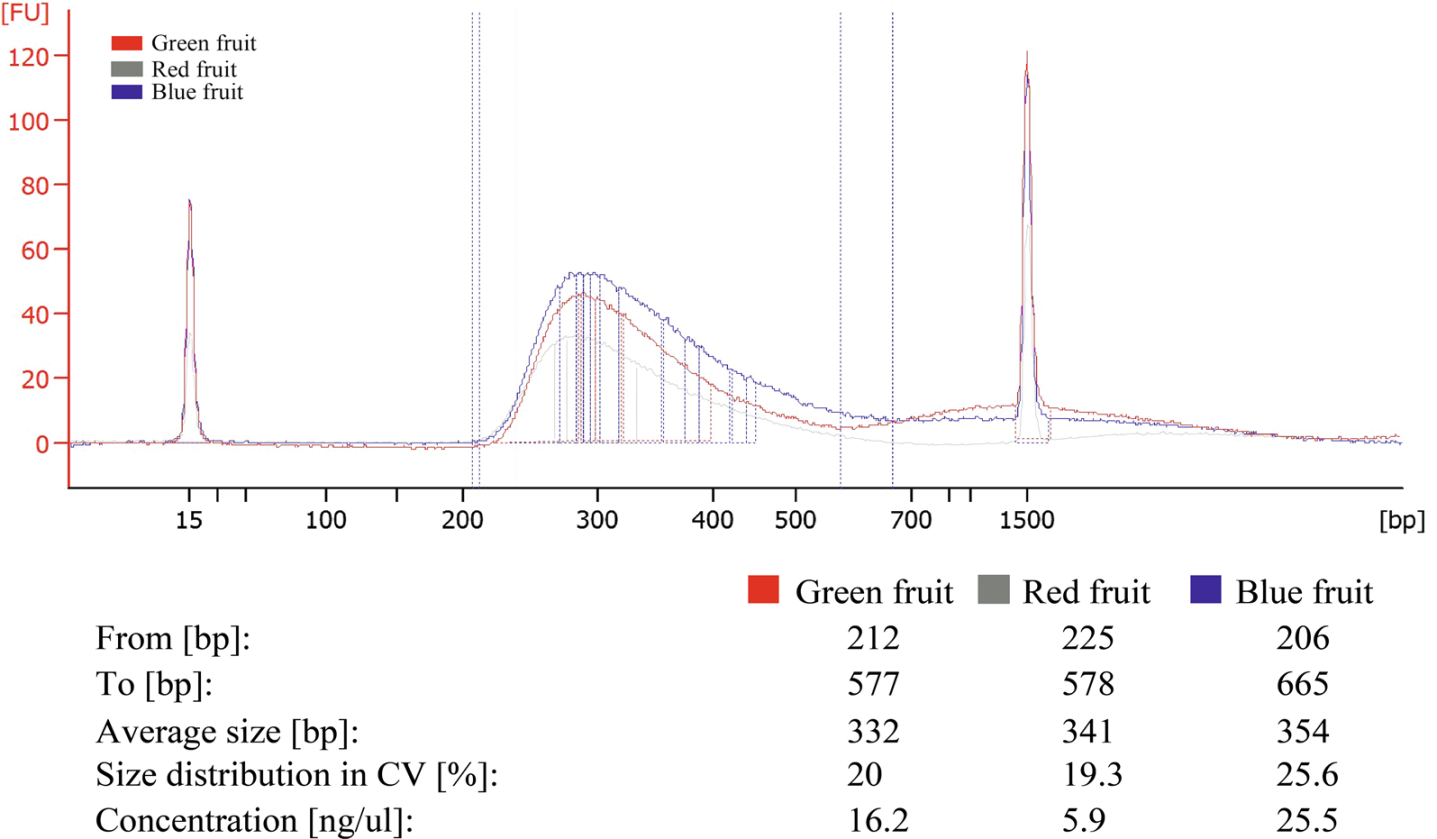
Electrophoretogram of sequencing libraries. The* graph* shows the length distribution curves of sequencing libraries obtained with Illumina TruSeqTM RNA sample preparation kit (Low-Throughput protocol) according to manufacturer’s protocol. The length of DNA fragments was between 200 and 700 pb. Curves were generated on a 2100 bioanalyzer using DNA 1000 chip (Agilent Technologies). For each sample three biological replicates were performed

Transcriptomics using next-generation sequencing requires high concentration and quality as clean RNA material. In order to test if the RNA obtained by our new method is suitable for this technology, we performed a transcriptome analysis for different fruit tissues. Three Maqui berry libraries (green fruit, red fruit and blue fruit) were prepared using the TruSeq™ RNA Sample Preparation Kits v2 (Illumina^®^). As this kit is optimized for 0.1–1 μg total RNA, the concentrations obtained with our method were enough for library preparation. The quality of these libraries was assessed with a DNA 1000 kit in a 2100 Bioanalyzer (Agilent Technologies). As shown in Fig. 2, the different libraries had a range of concentrations that varied between 5.9 and 25.5 ng/µl. Despite this concentration disparity, they all showed a relatively uniform fragment average size (332–354 bp) and size distribution (19.3–25.6 %). The libraries were ready to be sequenced using next-generation sequencing platform and was achieved a high quality de novo sequence assembly.

## Conclusions

In summary, a CTAB-based RNA isolation procedure was modified in order to isolate high quality RNA from different Maqui berry tissues.

The modified protocol can be used reliably to isolate high quality RNA from all stages of Maqui fruit development. Additionally, the RNA obtained by this protocol can also be used in a wide variety of applications, such as real time PCR, cDNA library construction, RNA-Seq, all of which require a high RNA quality. Our adapted method showed a great improvement in purity, integrity and yield of RNA. This method provides a new approach that could be used in other plants with extremely high levels of polyphenols, especially anthocyanins.

## Methods

### Sampling

All samples were collected from the same wild Maqui berry tree grown in Angol (37°50′S, 72°42′W, Araucanía, IX Region, Chile). As shown in Fig. 1, three consecutive fruit ripening stages were sampled and their age was measured as days after flowering (DAF). The date of flowering (day 0) was recorded as the day on which 50 % of the inflorescence was flowering. Samples corresponding to young fruits (green, 30 DAF), semi-ripe fruits (red, 60 DAF), and ripe fruits (blue, 90 DAF), were randomly collected from several clusters of the same tree. Plant material was frozen in liquid nitrogen and subsequently stored at −80 °C for further processing.

### Solutions and reagents

All the solutions were prepared in 0.1 % diethylpyrocarbonate (DEPC)-treated water and autoclaved or freshly used. The extraction buffer contained the following reagents: 2 % CTAB, 4 % polyvinylpyrrolidone (PVP-40), 100 mM Tris–HCl (pH 8.0), 25 mM EDTA and 2.0 M NaCl. Mix and sterilize in an autoclave. Filter-sterilized spermidine and β-mercaptoethanol should be added to a final concentration of 0.5 g/l and 4 %, respectively, just before use. SSTE buffer: 1.0 M NaCl, 0.5 % SDS, 10 mM Tris–HCl (pH 8.0), 1 mM EDTA (pH 8.0). Mix and do not autoclave. Other solutions and reagents used in RNA isolation were the following: Chloroform-isoamyl alcohol (24:1, v/v); phenol/chloroform-isosmylalcohol (25:24:1,v/v); pH 4.3-equilibrated phenol solution (Sigma, catalog number P4682); 10 M lithium chloride; absolute ethanol; 70 % ethanol; diethylpyrocarbonate-treated and autoclaved distilled water. All non-disposable plastic materials were treated with diethylpyrocarbonate and autoclaved. Glass material, the mortar and pestle were treated for 180 °C for at least 4 h.

### RNA extraction protocol

Our RNA extraction protocol from Maqui berry was adapted from Jaakola et al. (2001). Before the procedure, 7.500 µl of extraction buffer were added to a 10 ml tube. This tube was heated for at least 10 min at 65 °C in a water bath. It was important to ensure that the extraction buffer was noticeable hot before using it for tissue grinding.

1.5 g of fresh tissue were ground to fine powder with a mortar and pestle, and liquid nitrogen. The ground material was quickly transferred to 10 sterile 1.5 ml microcentrifuge tubes. The tubes were kept on ice until the next step. 750 μl preheated extraction buffer were added to each sample and completely mixed by inversion. The tubes were incubated for 10 min at 65 °C. The samples were agitated with a vortex for a few seconds during the incubation time. The tubes were subsequently centrifuged at 10,000×*g* (RCF) for 10 min at 4 °C. The supernatants were removed and added to new pre-chilled Eppendorf tubes. An equal volume of chloroform:IAA was added, mixed and centrifuged at 15,700×*g* (RCF) for 10 min at 4 °C, in order to separate the phases. The supernatant was recovered, 1/4 volume of 10 M LiCl was added and mixed gently. RNA was precipitated at −80 °C overnight and subsequently centrifuged at 18,000×*g* (RCF) for 20 min at 4 °C. The supernatant was decanted by pressing the tubes against a paper towel. The pellet was washed with 500 µL of 70 % ice-cold ethanol. Then, the tubes were centrifuged briefly and the ethanol was decanted. For ripe fruits, this washing step was repeated at least twice, until the dark color disappeared.

The final RNA pellet was dissolved in 100 µl SSTE buffer. The 10 dissolved RNA samples were pooled into two Eppendorf tubes. For samples dissolved slowly, the tubes were briefly heated at 65 °C. An equal volume of phenol:chloroform:IAA (25:24:1) was added to the sample and shaken vigorously, then incubated on ice for 5 min and centrifuged at 18,000×*g* (RCF) for 20 min at 4 °C. The supernatant was carefully transferred to a new tube and an equal volume of chloroform:IAA was added. Then, samples were centrifuged as described above. The upper aqueous phase was collected carefully to a new tube and precipitated by adding an equal volume of absolute ethanol at −20 °C for 2 h or at −80 °C for 30 min. Samples were centrifuged at 18,000×*g* (RCF) for 15 min at 4 °C, the supernatants were discarded and the resulting pellets were carefully washed twice with chilled 70 % ethanol. The final pellets were air dried and then dissolved in 200 μl DEPC-treated water. In order to eliminate any genomic DNA contamination that could interfere with gene expression studies, the total RNA obtained by our method was subjected to an exhaustive DNase I digestion (Ambion), according to the manufacturer's instructions. 2 μl RNA samples were used for quality assessment. The rest was stored at −80 °C.

### Determination of RNA concentration and purity

RNA concentration and purity was assessed by spectrophotometer. The concentration of RNA was quantified in the Infinite^®^ 200 NanoQuant spectrophotometer (Tecan). The RNA was diluted in autoclaved double distilled water to make dilution factor of 10, i.e. 1 µl RNA sample +9 µl autoclaved double distilled water. The quantification of RNA was done in triplicates. The O.D values were taken 260 and 280 nm. RNA concentration and the 260/280 ratios were delivered by the instrument.

### Determination of RNA integrity

RNA integrity was reported as the RIN based on the entire electrophoretic RNA sample trace, produced using the Agilent 2100 Bioanalyzer. The concentration and integrity of total RNA were determined by assaying 1 µl of total RNA using Agilent’s 2100 bioanalyzer with the Plant RNA Pico chip assay in accordance with the manufacturer’s instructions (Agilent Technologies, Santa Clara, CA, USA). The Bioanalyzer uses electrophoretic technology on a chip to separate RNA fragments by size, which are read by laser induced fluorescence and translated into gel-like bands and peaks (electropherograms) showing the distribution and relative amounts of RNA of different sizes. Concentration (ng/µl) of RNA was determined by comparing the sample with a standard. It measured RNA integrity using two metrics: the ratio of the large (26S) to small (18S) ribosomal RNA subunits (26S/18S) and the RIN.

### RNA-Seq library construction

Three Maqui berry libraries (green fruit, red fruit and blue fruit) were prepared using the TruSeqTM RNA Sample Preparation Kits v2 (Illumina^®^), according to the manufacturer’s instructions. Briefly, 1 μg of total RNA was used as a starting material in order to isolate mRNA using Oligo(dT) beads. The poly(A) RNA was subsequently fragmented with divalent cations under elevated temperatures. These short fragments were used as templates for first-strand cDNA synthesis, producing single-stranded DNA copies from the fragmented RNA. After second-strand cDNA synthesis, the double-stranded DNA was subjected to end repair, and the 3′ends were adenylated. Finally, universal adapters were ligated to the cDNA fragments, and enrichment of those DNA fragments was performed using 15 cycles of PCR to produce the final sequencing library. Subsequently, the libraries were validated using DNA 1000 chip (on Agilent Technologies 2100 bioanalyzer). The samples were pooled together in equal concentrations in one pool and running on an Illumina MiSeq™ System for a 300 cycles of paired-end sequencing.

## Abbreviations

CTAB: hexadecyltrimethyl ammonium bromide
PVP: polyvinylpyrrolidone
RIN: ribosomal integrity number
